# Molecular mechanisms and therapeutic possibilities of short-chain fatty acids in posttraumatic stress disorder patients: a mini-review

**DOI:** 10.3389/fnins.2024.1394953

**Published:** 2024-06-03

**Authors:** Pavlo Petakh, Khrystyna Duve, Valentyn Oksenych, Payam Behzadi, Oleksandr Kamyshnyi

**Affiliations:** ^1^Department of Biochemistry and Pharmacology, Uzhhorod National University, Uzhhorod, Ukraine; ^2^Department of Microbiology, Virology, and Immunology, I. Horbachevsky Ternopil National Medical University, Ternopil, Ukraine; ^3^Department of Neurology, I. Horbachevsky Ternopil National Medical University, Ternopil, Ukraine; ^4^Broegelmann Research Laboratory, Department of Clinical Science, University of Bergen, Bergen, Norway; ^5^Department of Microbiology, Shahr-e-Qods Branch, Islamic Azad University, Tehran, Iran

**Keywords:** gut microbiome, stress, posttraumatic stress disorder, probiotic, SCFA, acetate, propionate, butyrate

## Abstract

This mini-review explores the role of short-chain fatty acids (SCFAs) in posttraumatic stress disorder (PTSD). Highlighting the microbiota-gut-brain axis, this study investigated the bidirectional communication between the gut microbiome and mental health. SCFAs, byproducts of gut microbial fermentation, have been examined for their potential impact on PTSD, with a focus on molecular mechanisms and therapeutic interventions. This review discusses changes in SCFA levels and bacterial profiles in individuals with PTSD, emphasizing the need for further research. Promising outcomes from clinical trials using probiotics and fermented formulations suggest potential avenues for PTSD management. Future directions involve establishing comprehensive human cohorts, integrating multiomics data, and employing advanced computational methods, with the goal of deepening our understanding of the role of SCFAs in PTSD and exploring microbiota-targeted interventions.

## 1 Introduction

Posttraumatic stress disorder (PTSD) manifests as a set of distinct symptoms arising from exposure to, or observation of, highly distressing events carrying a genuine risk of death, actual death, or severe injury (Gradus and Galea, 2022).

In line with the Diagnostic and Statistical Manual of Mental Disorders (DSM-5), PTSD is characterized by four symptom clusters: re-experiencing, avoidance, negative alterations in cognition and mood, and alterations in arousal and reactivity (American Psychiatric Association D, Association AP, 2013; Pai et al., 2017). This chronic mental health condition can instigate persistent feelings of fear, disarray, or panic following an encounter with or witnessing a traumatic incident (Panagioti et al., 2015; Pai et al., 2017). In addition to the main symptoms of PTSD, patients also experience PTSD-related symptoms such as depression, anxiety, insomnia, and circadian rhythm disturbances (Short et al., 2020; Kyzar et al., 2021).

Recent research has increasingly focused on the intricate interplay between the gut microbiome and PTSD, revealing the potential role of the gut-brain axis in influencing mental health (Gautam et al., 2015; Ke et al., 2023; Petakh et al., 2024a).

The gut microbiome, a diverse community of microorganisms residing in the gastrointestinal tract, plays a crucial role in maintaining physiological balance and impacting various aspects of human health (Thursby and Juge, 2017). Short-chain fatty acids (SCFAs), metabolic byproducts of gut microbial fermentation, have garnered attention for their potential involvement in mental health conditions, including PTSD (van de Wouw et al., 2018).

This mini-review delves into the molecular mechanisms and therapeutic possibilities of SCFAs in the context of PTSD. As we explored the intricate connections between the gut microbiota, SCFAs, and PTSD, we aimed to provide insights into the changes in SCFA levels and the profiles of bacteria producing them in individuals affected by PTSD. Additionally, we will discuss potential therapeutic interventions, drawing from both clinical trials and emerging research.

By examining the current state of knowledge on SCFAs and their impact on PTSD, this mini-review aims to contribute to the evolving understanding of the gut–brain axis and open avenues for novel approaches in the treatment and management of PTSD. Through a comprehensive exploration of the molecular underpinnings and therapeutic potential of SCFAs, we aspire to elucidate promising directions for future research and clinical applications in the field of mental health.

## 2 Short-chain fatty acids: overview

Among the myriad of metabolites generated by the microbiome, SCFAs are significant components. Acetate (C2), propionate (C3), and butyrate (C4) are the primary SCFAs, collectively representing a substantial portion. Acetate predominates, constituting approximately 60% of the SCFAs, while propionate and butyrate each contribute approximately 20%. Although SCFAs are prominent, they do not encompass the entirety of microbiota-derived metabolites. Other metabolites, including various lactate isomers, valerate, isobutyrate, isovalerate, and secondary bile acids, also exist, albeit at lower concentrations (Cait et al., 2018). Secondary bile acids are formed by enzymatic modifications of primary bile acids by bacteria present in the colon, where they serve as substrates for microbial metabolism (McMillin and DeMorrow, 2016). For example, secondary bile acids can activate bile acid receptors located in different organs, participating in signaling pathways from the gut to other organs. This hypothesis is supported by the presence of bile acid receptors in the liver, brain, and muscles, suggesting a bile acid-gut-organ axis (Mohanty et al., 2024).

SCFAs exert their effects in part through free fatty acid receptors (FFARs), with FFAR2 (also known as GPR43) and FFAR3 (known as GPR41) being key players. These G protein-coupled receptors are found on various cells, including neurons, colonocytes, pancreatic cells, adipocytes, and others (Yang et al., 2020). Acetate and propionate predominantly activate FFAR2, while butyrate influences FFAR3 (Brown et al., 2003). These receptors have implications for inflammation modulation, energy consumption in neurons, insulin secretion, and enteroendocrine function (Prentice et al., 2019; Jiao et al., 2021; Zou et al., 2021).

These receptors are located on the apical membrane of the colon epithelium, where luminal SCFAs activate them to initiate intracellular second messenger signaling cascades (Smith et al., 2013; McKenzie et al., 2017). Their activation triggers signaling pathways involving the Gαs and Gβγ subunits, leading to ERK activation, a reduction in cellular cyclic adenosine monophosphate (cAMP) levels, and an increase in intracellular Ca^2+^ concentrations. This, in turn, serves as a secondary messenger that initiates various biological responses and downstream signaling cascades, including protein phosphorylation and alterations in cellular behavior (Le Poul et al., 2003; Gaidarov et al., 2013; Park et al., 2021). Furthermore, the interaction of GPCRs with β-arrestin (β-ARR) also stimulates the activation of Gαs and Gβγ subunits, thereby modulating physiological processes such as chemotaxis, apoptosis, proliferation, differentiation, and gene expression *in vivo* (O’Hayre et al., 2017; Böttke et al., 2020). Notably, while the FFAR3 receptor has been reported to signal exclusively via the Gi protein family, FFAR2 has been found to activate G proteins from both the Gi and the Gq families, but induces signaling in a Gαi/o/q/11- and β-arrestin-independent fashion, which has been shown to be mediated via Gα12/13 proteins (Grundmann et al., 2021). Following their uptake by colonocytes, SCFAs proceed into the mitochondria’s citric acid cycle, where they participate in ATP synthesis, providing essential energy for cellular functions (Schönfeld and Wojtczak, 2016).

Their passage through the epithelium is crucial for reaching the serosal side and influencing immune cells in the lamina propria (Kaji et al., 2015; Stumpff, 2018). Effective transport mechanisms are essential for luminal entry and transcellular transport, without which SCFAs cannot exert intracellular effects on colon epithelial cells or impact mucosal immune cells (Binder, 2010). Given the physiological colonic pH, SCFAs predominantly exist in anionic forms, hindering simple diffusion. Consequently, ionized SCFAs are anticipated to require carrier-mediated absorption pathways. Numerous studies have highlighted evidence supporting the existence of such pathways for ionized SCFA absorption. Thus, the presence of SCFA transporters within colon epithelial cells significantly influences the beneficial effects of SCFAs on the host. Notably, transporters such as H + -coupled (e.g., monocarboxylate transporter MCT1/4) and Na + −coupled (e.g., sodium-coupled monocarboxylate transporter SMCT1/2) transporters facilitate the transfer of SCFAs into colon epithelial cells, playing pivotal roles in SCFA absorption and utilization within the intestine (Sivaprakasam et al., 2017; Holota et al., 2019).

As evidenced by experiments in cell cultures, these effects may be facilitated by the presence of abundant monocarboxylate transporters (MCTs) on endothelial cells (Mitchell et al., 2011). Studies in rats have shown that following injection into the carotid artery, butyrate exhibits the highest brain uptake, followed by propionate and acetate (Kekuda et al., 2013; Vijay and Morris, 2014). A study reported that the human brain has an average content of 17.0 pmol/mg of tissue for butyrate and 18.8 pmol/mg of tissue for propionate. When the data are recalculated to account for brain water, which makes up approximately 75% of the brain’s weight, the levels of both SCFAs increase. Specifically, the levels of butyrate and propionate are approximately 21 μM and 23 μM, respectively, which are similar to the levels found in plasma (Bachmann et al., 1979). PET imaging experiments in rats indicated that a small percentage of intravenously infused acetate was rapidly taken up by the brain, with slightly lower uptake observed after colonic infusion (Kim et al., 2013). However, studies in primates and humans using labeled SCFAs have shown minimal brain uptake, suggesting that despite their ability to cross the BBB, SCFAs are not substantially absorbed by the brain (Seltzer et al., 2004).

SCFAs also exert their effects by inhibiting histone deacetylases (HDACs), which are enzymes responsible for removing acetyl groups from histone and nonhistone complexes, thereby regulating gene expression. Inhibition of HDACs by SCFAs leads to increased histone acetylation, resulting in relaxation of chromatin structure and enhanced accessibility of certain genes to transcription factors, ultimately promoting their expression (Friedrich et al., 2019). This mechanism is particularly relevant in inflammatory diseases, where HDAC inhibitors have shown potent anti-inflammatory activity (Elfiky et al., 2022). SCFAs, which are produced by intestinal microorganisms, play a significant role in this process, especially during intestinal barrier repair and in the regulation of metabolic diseases (Bose et al., 2014; Li et al., 2018; Vizioli et al., 2020). In intestinal epithelial cells (IECs), a specific HDAC known as HDAC3 regulates histone acetylation and integrates signals from the gut microbiota to maintain intestinal homeostasis (Zhang et al., 2020; Zhou et al., 2021).

SCFAs confer several health benefits, particularly through the upregulation of tight junction (TJ) proteins and reinforcement of the mucus layer, with butyrate notably contributing to these effects (Higashimura et al., 2015; Shimizu et al., 2019). Additionally, SCFAs contribute to the prevention of colon cancer by inducing the differentiation and apoptosis of colonic cells (Weitkunat et al., 2016). SCFAs, particularly acetate, play a role in appetite regulation and human metabolism. They may reduce appetite, body weight, and liver steatosis while modulating glucose and lipid metabolism (Zhao et al., 2017; Bartolomaeus et al., 2019; Hsu et al., 2019; Yu et al., 2021). Increased levels of butyrate and propionate are associated with reduced blood pressure and plasminogen activator inhibitor-1 (PAI-1) levels in the context of cardiometabolic health (Xu et al., 2022). SCFAs have notable immunomodulatory effects, affecting both innate and adaptive immunity. These compounds reduce neutrophil activity, inhibit inflammatory cell chemotaxis, enhance regulatory T-cell activity, and suppress gut inflammation (Li et al., 2018, 2021; Liu et al., 2021).

The primary source of bacteria responsible for SCFA production is the intestinal microbiota, which engages in the fermentation of dietary fiber and resistant starches to generate SCFAs. Intriguingly, the concentration of SCFAs exhibits dynamic changes throughout our lifespan, and these variations seem to correlate with shifts in the composition of the gut microbiome, which itself undergoes alterations across different stages of life (Li et al., 2019; Meng et al., 2019). It is important to highlight that many internal and external factors, such as diet, drug use, and exercise, play significant roles in determining the quantity of SCFAs produced within the intestines. These changes in diet, for example, in turn, affect the availability of substrate sources for bacteria engaged in SCFA production (Sanna et al., 2019).

## 3 The microbiota-gut-brain axis

A growing body of scientific research highlights the existence of a microbiota–gut–brain axis linking the gut microbiota to mental health (Adan et al., 2019; Bastiaanssen et al., 2019; Behzadi et al., 2024; Petakh et al., 2024b). This axis involves bidirectional communication between the brain, gut, and gut microbiome through various pathways, including nervous, endocrine, and immune signaling pathways (Cryan and Dinan, 2012).

**Figure 1:**
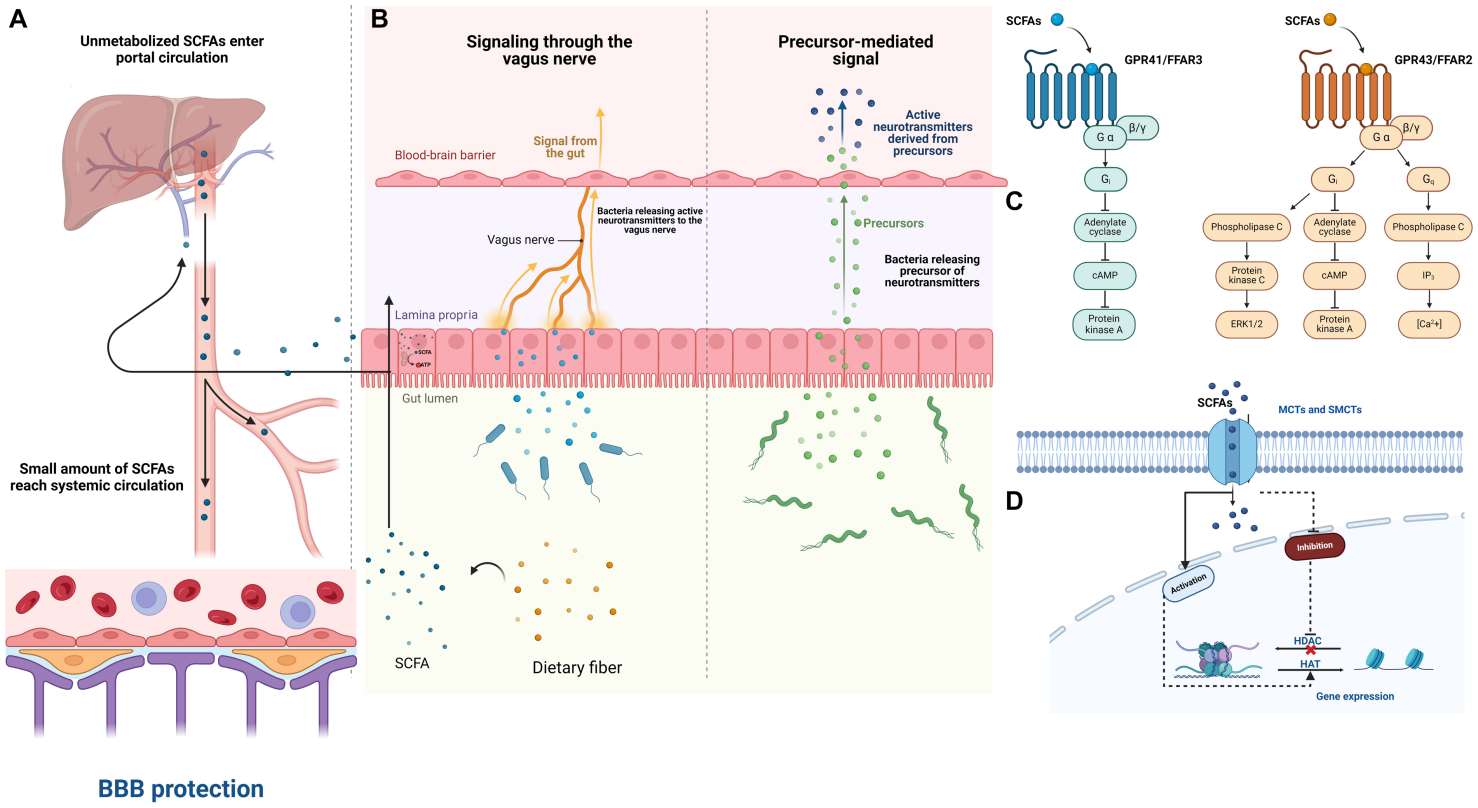
The impact of SCFAs on the Microbiota–Gut–Brain axis in PTSD patients. **(A)** The breakdown of dietary fiber by beneficial gut bacteria in the colon results in the production of short-chain fatty acids (SCFAs). These SCFAs are quickly absorbed by colon cells through various mechanisms and are partially utilized for cellular energy production. Any remaining SCFAs enter the bloodstream and are utilized as energy by liver cells or incorporated into metabolic processes. Consequently, only a limited quantity of SCFAs generated in the colon enter systemic circulation. **(B)** The microbiota can influence the brain through various mechanisms, such as microorganism-produced neurotransmitters affecting the brain via the vagus nerve and its afferent nerves. Additionally, neurotransmitter precursors may pass through the blood–brain barrier and be converted into active neurotransmitters. **(C)** SCFAs potentially impact interactions between the microbiota, gut, and brain by communicating with the host through the free fatty acid receptors (FFARs) FFAR2 and FFAR3. **(D)** Intracellular short-chain fatty acids (SCFAs) also play a role in histone acetylation and deacetylation, which affects gene transcription. This can occur by inhibiting histone deacetylases (HDACs), leading to more active chromatin for transcription, or by enhancing the activity of histone acetyltransferases (HATs), thus promoting acetylation. These processes may occur not only in colonocytes but also in any cell accessible to SCFAs within a tissue, either indirectly through FFARs or directly through monocarboxylate transporters (MCTs) and sodium-dependent monocarboxylate transporters (SMCTs).

Both chronic and acute stressors can alter the composition of gut bacteria in various areas and environments, including the interior (lumen) and the lining (mucosal) of the gut (Bailey et al., 2011; Galley et al., 2014; Madison and Kiecolt-Glaser, 2019). Chronic stress has also been linked to lasting changes in the gut microbiota in animal models. These changes can lead to variations in alpha diversity, which seem to depend on the specific characteristics of the stressors (Geng et al., 2019). For instance, research has shown that students under academic stress have a gut microbiota with a lower abundance of beneficial bacteria (Knowles et al., 2008). Additionally, recent evidence has demonstrated an imbalance in the microbiota of frontline healthcare workers who experienced psychological stress while working during the COVID-19 pandemic (Gao et al., 2022). Both acute and chronic stress can lead to structural and functional alterations in the human gut microbiota (Ma et al., 2023; Figure 1).

Rodent models have shown that the gut microbiome affects stress-related neurocircuitry, feeding behavior, and obesity through neuroimmune-neuroendocrine pathways (Bastiaanssen et al., 2021). Concerning PTSD, both human studies and animal experiments suggest a brain-gut connection, with the gut microbiota influencing amygdala development and response, which is relevant because the amygdala plays a central role in stress and PTSD (Buffington et al., 2016; Gorecki et al., 2019; Chen et al., 2020; Ousdal et al., 2020).

PTSD-related symptoms such as anxiety, depression, and insomnia are also connected with the gut microbiota (Madison and Kiecolt-Glaser, 2019; Nikolova et al., 2021). Recently, studies have shown a connection between intestinal dysbacteriosis and stress as well as anxiety (Kelly et al., 2016; Burokas et al., 2017). Anxiety-related disorders, such as generalized anxiety disorder (GAD), are associated with gut dysbiosis (Ritchie et al., 2023). In cases of depression and anxiety, there is a notable increase in the number of gut bacteria across various taxonomic levels, including 15 genera and 18 species (Jiang et al., 2018; Ritchie et al., 2023). Patients with GAD exhibit a decrease in the abundance of bacteria that produce SCFAs and an increase in the abundances of *Escherichia-Shigella*, *Fusobacterium*, and *Ruminococcus gnavus*. These modifications persisted even after the alleviation of GAD (Ritchie et al., 2023). The severity of anxiety is correlated with the abundance of *Escherichia-Shigella* and *Bacteroides* in patients with active GAD (Ritchie et al., 2023). Distinct patterns have been observed in the gut microbiome related to GAD and depression. Specifically, interactions between C-reactive protein (CRP) and bacterial families indicate possible links to these conditions. For depressive symptoms (measured by the PHQ-9), notable associations included the bacterial families Ruminococcaceae, Akkermansia, and Acidaminococcaceae. For GAD (measured by GAD-7), significant relationships were found with the bacterial orders Bacteroidales, Selenomonadales, and Clostridiales. The genus *Holdemanella* was significantly associated with both GAD and depression. These associations suggest that inflammation and the gut microbiota may play a role in mental health (Chen et al., 2021).

In their study, Zheng et al. discovered that individuals suffering from depression exhibited a greater proportion of Actinobacteria and Firmicutes, while the abundance of Bacteroidetes decreased (Zheng et al., 2016). Jiang et al. reported that patients with depression exhibited significantly greater Shannon index scores for intestinal microbiota than did patients in the control group (Jiang et al., 2015). The levels of Bacteroidetes, Proteobacteria, and Actinobacteria were significantly greater, while the level of Firmicutes was significantly lower in the active-MDD (Major Depressive Disorder) and responsive-MDD groups than in the healthy controls. Furthermore, there was a negative correlation between the abundance of *Faecalibacterium* and the severity of depression in the patients. These findings suggest that depression may be associated with a predominance of potentially harmful bacterial groups or a reduction in beneficial bacterial genera. Furthermore, a separate study revealed a reduction in the prevalence of intestinal *Bifidobacteria* and *Lactobacilli* among individuals suffering from depression (Aizawa et al., 2016). Moreover, Voigt et al. reported that circadian disorganization disrupts the composition of the intestinal microbiota (Voigt et al., 2014). Emerging evidence strongly indicates notable links between circadian rhythm disturbances and mental health conditions, such as PTSD (Agorastos et al., 2014; Olff et al., 2019). Furthermore, researchers have initiated investigations into the interplay between the circadian system and mental health (Walker 2nd et al., 2020). For instance, individuals who develop PTSD often experience more disrupted sleep patterns following a traumatic event (Tsanas et al., 2020). Conversely, sleep has been found to play a protective role in preventing the formation of intrusive memories (Kleim et al., 2016). The variability in the findings of these studies may be attributed to factors such as the size of the sample, ethnic origin, diet habits, and use of antidepressant medication among the participants.

Changes in the microbiota may impact both the nervous and immune systems, diminishing an individual’s ability to manage psychological and physical stress and rendering them more susceptible to stressful situations. Nevertheless, certain probiotics, such as *Lactobacillus*, *Bifidobacterium*, and *Enterococcus*, can modulate emotional and cognitive parameters by influencing the enteric nervous system and the immune system (Plaza-Díaz et al., 2017). This modulation can enhance stress resilience and produce an anti-anxiety effect.

Depression and insomnia often coexist, with many patients reporting poor sleep quality as a primary concern (Seow et al., 2016). Recent research has suggested a connection between the gut-brain axis (GBA) and the concurrent occurrence of depression and insomnia. Microbial activity in the gut leads to the production of various substances, including neurotransmitters like serotonin (5-HT), dopamine (DA), and GABA, metabolites such as SCFAs and melatonin, and also influences cytokine levels (Petra et al., 2015). These compounds influence both the enteric nervous system (ENS) and the central nervous system (CNS) either directly or indirectly by interacting with enteroendocrine cells (Ridaura and Belkaid, 2015).

Certain bacteria, such as *Lactobacillus* and *Bifidobacterium*, can produce GABA, which is linked to mood regulation. Studies on animals have shown that administering *Lactobacillus rhamnosus* (JB-1) leads to reduced anxiety and depression-like behaviors, along with changes in GABA receptor expression in the brain, akin to the effects of benzodiazepines (Bravo et al., 2011; Cryan et al., 2019). However, these effects were not observed in animals with severed vagus nerves, indicating the importance of this nerve pathway in gut–brain communication (Schulze et al., 2014). Lactobacilli can produce SCFAs by fermenting carbohydrates. This involves the use of pyruvate from glycolysis or, under heterofermentative conditions, the phosphoketolase pathway (Pessione, 2012).

In summary, there is a two-way relationship linking the gut microbiome with both sleep patterns and depression. Inflammation and hormonal changes are pivotal in this dynamic. Initially, disruptions in the body’s circadian rhythms, sleep disturbances, and depressive symptoms impact the metabolism of resident gut bacteria, leading to alterations in their composition (Thaiss et al., 2014). Gut bacteria can influence the integrity of epithelial cell junctions, thereby regulating intestinal permeability and safeguarding the intestinal barrier (Yarandi et al., 2016).

A study revealed that 4 mmol/L butyrate increased the relative expression of occludin and ZO-1 mRNA in IPEC-J2 cells and claudin-1 mRNA in rat cdx2-IEC cells (Ma et al., 2012; Wang et al., 2012). This led to reduced intestinal permeability and greater villus height in the mice. Tong et al. (2016) reported that propionic acid elevated the levels of intestinal tight junction proteins such as ZO-1, occludin, and cadherin, which supported improved intestinal function (Tong et al., 2016). SCFAs were also shown to activate the AMPK pathway, thereby boosting the expression of ZO-1 in intestinal epithelial cells (Voltolini et al., 2012; Tong et al., 2016). This action enhances transepithelial electrical resistance (TEER) and contributes to the protection of intestinal barrier integrity. Huang et al. (2015) reported that sodium butyrate considerably increased the occludin protein in the jejunum and colon of weaned piglets, decreasing diarrhea through decreased intestinal permeability (Huang et al., 2015). Studies have also shown that butyrate can control the growth, apoptosis, and differentiation of the gastrointestinal epithelium (Miao et al., 2016). Butyrate improved barrier function by positively regulating the expression of claudin-1, ZO-1, and occludin in Cdx2-IECs and Caco-2 cells (Wang et al., 2012). This led to an increase in TEER. The effect of butyrate on the epithelial barrier may be due to the upregulation of TJ proteins through the activation of AMP-activated protein kinase (Peng et al., 2009).

When this barrier breaks down, harmful bacteria and their byproducts may infiltrate mesenteric lymph tissue, inciting inflammatory immune responses and activating the vagus and spinal afferent nerves (Hsiao et al., 2013) and further impacting the central nervous system, potentially contributing to or exacerbating insomnia and depression (Yi and Li, 2012).

## 4 Differences in SCFA and bacterial profiles in PTSD patients

Emerging research has focused on the gut microbiota, recognizing its potential role in modulating stress responses and mental health outcomes. Here, we delve into the key findings from recent studies investigating the relationship between gut microbiota composition and PTSD severity.

Hemmings et al. (2017) and Malan-Muller et al. (2022) both conducted studies within South African cohorts, shedding light on distinct microbial signatures associated with PTSD. Hemmings et al. identified *Actinobacteria*, *Lentisphaerae*, and *Verrucomicrobia* as potential discriminators between PTSD-diagnosed individuals and trauma-exposed controls (Hemmings et al., 2017). Notably, Malan-Muller et al. reported that increased levels of certain genera, such as *Mitsuokella*, *Odoribacter*, *Catenibacterium*, and *Olsenella*, were linked to worsening PTSD (Malan-Muller et al., 2022).

A study by Bajaj et al. (2019) examined male cirrhotic veterans who had been in combat. They found that the microbiota was less diverse, had more pathobionts (*Enterococcus* and *Escherichia/Shigella*), and had different amounts of autochthonous taxa (*Lachnospiraceae* and *Ruminococcaceae*) in people who had PTSD related to combat compared with non-PTSD combat-exposed patients. Importantly, despite adjusting for confounding factors, functional alterations persisted, suggesting a direct link between gut dysbiosis and PTSD pathogenesis (Bajaj et al., 2019).

Ella Levert-Levitt and colleagues discovered that a distinct microbiota pattern characterized by reduced levels of certain bacteria, such as sp_HMT_914, sp_HMT_332, and sp_871, along with Noxia, was associated with the severity of PTSD symptoms, including intrusive thoughts, heightened arousal, and reactivity, as well as other psychological issues, such as anxiety, hostility, memory problems, and unexplained pain (Levert-Levitt et al., 2022).

Feldman et al. (2022) examined mother–child pairs and found that microbial trauma profiles, especially those from the genera *Dialister* and *Veillonella*, were linked to trauma-related phenotypes (Feldman et al., 2022). Finally, Yoo et al. (2023) investigated the gut microbiota of firefighters, revealing associations between specific bacterial taxa (*Lachnospiraceae blautia*, *Lachnospiraceae coprococcus*, and *Alistipes onderdonkii*) and PTSD symptom severity. Interestingly, they identified interactions between bacterial abundance and PTSD severity scores, offering insights into potential microbial biomarkers for PTSD risk assessment and personalized interventions (Table 1; Yoo et al., 2023).

**Table 1:** Studies investigating the microbiome in PTSD populations.

Study	Population	Sample types	Sequencing platform	Main findings
Hemmings et al. (2017)	South African cohort (*n* = 30)	Fecal sample	16S rRNA gene sequencing	Three phyla (Actinobacteria, Lentisphaerae, and Verrucomicrobia) can distinguish those with PTSD from those who were trauma exposed but did not have a PTSD diagnosis. Decreased total abundance of these taxa was associated with higher PTSD CAPS scores. The relative abundance of Actinobacteria and Verrucomicrobia was associated with childhood trauma scores.
Bajaj et al. (2019)	Combat-exposed male cirrhotic veterans (*n* = 93)	Fecal sample	16S rRNA gene sequencing	Combat-related PTSD is associated with lower microbial diversity, higher pathobionts (Enterococcus and Escherichia/Shigella), and lower autochthonous taxa composition (Lachnospiraceae and Ruminococcaceae). Despite accounting for prior hepatic encephalopathy, psychoactive drug use, or model for end-stage liver disease score, functional alterations were observed.
Malan-Muller et al. (2022)	South African cohort (*n* = 137)	Fecal sample	16S rRNA gene sequencing	The relative abundance of a consortium of four genera (Mitsuokella, Odoribacter, Catenibacterium, and Olsenella) was higher in the PTSD group than in the trauma-exposed controls and correlated positively with PTSD severity.
Levert-Levitt et al. (2022)	Israeli veterans (*n* = 189)	Salivary vial collection kit	16S rRNA gene sequencing	Decreased levels of the bacteria sp_HMT_914, 332, and 871 and Noxia were correlated with PTSD severity. Microbiota signature (decreased levels of sp_HMT_914, 332, and 871 and Noxia) was correlated with PTSD severity and other psychopathological symptoms. Education duration correlated with microbiota composition and adverse psychopathological measures. Air pollution was positively correlated with PTSD symptoms, psychopathological symptoms, and microbiota composition.
Feldman et al. (2022)	Mother–child dyads from Sderot, Israel (*n* = 148)	Fecal sample	16S rRNA gene sequencing	The genera Dialister and Veillonella were negatively and positively correlated with PTSD, respectively. This study provides causative evidence that the microbial trauma profile is at least partially responsible for the trauma-related phenotype.
Yoo et al. (2023)	30 participants, 15 healthy male, firefighters and 15 controls	Fecal sample	16S rRNA gene sequencing	An interaction between the abundance of certain bacteria taxa and total PCL-C score above 28 (indicating moderate to severe PTSD symptoms in firefighters) was observed. Positive correlations were found between Lachnospiraceae blautia, Lachnospiraceae coprococcus, and *Alistipes onderdonkii* with total PTSD symptom scores, while negative associations were found with Veillonellaceae megasphaera and *Bacteroides coprocola*.

*Lachnospiraceae* are SCFA-producing microorganisms (Socała et al., 2021). They have anti-inflammatory and modulatory effects on the intestinal mucosa, contributing to gut health. However, two studies have reported a significant decrease in this bacterial taxon, which might impact the levels of SCFAs.

## 5 Mechanisms of action: how SCFAs influence the CNS

SCFAs play a crucial role in promoting gut health by exerting various local effects. Specifically, they help maintain the integrity of the intestinal barrier and offer protection against inflammation within the intestine (Lewis et al., 2010).

Bacterial strains in the gut can modulate neurotransmitter levels, influencing microglial activation and cerebral functions. Gut microbiota-CNS communication involves the sympathetic branch of the autonomic nervous system, which encompasses sensory fibers capable of conveying signals from the gut to the CNS (Rhee et al., 2009; Kelly et al., 2017). SCFAs, which are bacterial metabolic byproducts, are considered key mediators of gut–brain communication, and altered SCFA production is observed in various neuropathologies (Tan et al., 2014).

In a cohort study from the Flemish Gut Flora Project, butyrate-producing bacteria, including Faecalibacterium and Coprococcus, were positively associated with higher quality of life indicators (Valles-Colomer et al., 2019). Conversely, those with major depressive disorder (MDD) tend to have increased levels of *Prevotella* and *Bifidobacterium*, which are associated with this condition and can be easily assessed for diagnostic and therapeutic purposes (Lin et al., 2017; Rong et al., 2019). The abundances of certain bacterial families, such as Lachnospiraceae and Ruminococcaceae, particularly genera such as *Roseburia* and *Blautia*, which are known for SCFA production from carbohydrate breakdown, are reduced in individuals with MDD relative to healthy individuals (Jiang et al., 2015). Recent research has also suggested the potential involvement of SCFAs in depression, with depressed individuals in Poland showing lower fecal propionate content and higher isocaproate levels (Skonieczna-Żydecka et al., 2018). These findings were associated with specific depressive symptoms, although the small sample size and the presence of other medical conditions among the participants need to be considered.

Persistent stress significantly increases the likelihood of developing neuropsychiatric disorders, with emerging research highlighting the crucial role of the microbiome-gut-brain axis in mediating this relationship (Ramirez et al., 2017; Cruz-Pereira et al., 2020). Recent clinical studies indicate that administering SCFAs directly to the colon may regulate the fundamental responsiveness of the hypothalamic–pituitary–adrenal (HPA) axis to psychosocial stress (Dalile et al., 2020). Animal studies have robustly demonstrated that prolonged stress disrupts the composition of the gut microbiome, while interventions targeting the microbiota can mitigate the physiological and neurological impacts of stress (Burokas et al., 2017). Several preclinical investigations revealed that supplementing mice subjected to extended psychosocial stress with acetate, butyrate, and propionate had positive effects on both behavior and stress-induced gut permeability (van de Wouw et al., 2018).

## 6 Psychobiotic-based interventions for PTSD

In 2013, the term “psychobiotics” was coined to describe “live microorganisms that, when consumed in sufficient quantities, confer health benefits to individuals experiencing psychiatric disorders” (Dinan et al., 2013).

A pilot randomized controlled trial investigated the feasibility, acceptability, and safety of supplementing veterans with clinically significant persistent postconcussive (PPC) and posttraumatic stress disorder (PTSD) symptoms using *Lactobacillus reuteri* DSM 17938. Thirty-one participants were randomized to either the probiotic or placebo group for 8 weeks. Probiotic supplementation demonstrated promising results, meeting feasibility and safety thresholds. While the decrease in plasma C-reactive protein (CRP) levels approached statistical significance, the placebo group exhibited a significantly greater increase in heart rate during the Trier Social Stress Test (TSST) math task. These findings suggest that *L. reuteri* DSM 17938 may have anti-inflammatory effects and impact stress responsiveness (Brenner et al., 2020).

A study involving ten combat veterans diagnosed with PTSD revealed that consistent intake of a fermented soy formulation (FSWW08) over a six-month period resulted in decreased symptoms such as anxiety, derealization/detachment, general infection, headache, loss of appetite, panic, upper gastrointestinal burning, and upper respiratory infection (Gocan et al., 2012). It is important to note that the study’s ability to establish causal relationships is limited due to the absence of a control group, the small sample size, potential selection biases, and reporting biases.

A study conducted on mice revealed that the administration of *L. rhamnosus* JB-1 as an early intervention (within 48 h of the traumatic event) for PTSD could have an adverse impact by enhancing fear. This suggests that the timing of stress and exposure to the bacterium may play a crucial role in determining its therapeutic effectiveness. Interestingly, the same study revealed comparable unfavorable outcomes when the mouse model received early treatment with sertraline, a frequently prescribed SSRI for PTSD (Liu et al., 2020). Another notable finding from C. Lowry’s group involving *Mycobacterium vaccae* is its potential to enhance fear extinction in PTSD-related paradigms. Specifically, immunization with *Mycobacterium vaccae* postfear conditioning led to enhanced fear extinction in rats (Hassell et al., 2019).

Another promising intervention for PTSD could be postbiotics, a concept formally proposed by Tsilingiri and Rescigno (2013). Postbiotics encompass any factors derived from the metabolic activity of probiotics or any released molecules that can directly or indirectly aid the host. These may include intentionally inactivated microbial cells with or without metabolites or cell components that contribute to health benefits, excluding pure microbial metabolites and vaccinations. In recent years, researchers have identified numerous distinct chemical types of postbiotics originating from bacterial cells, both within and outside the host organism. Postbiotics hold significant potential for future therapeutic applications (Lou et al., 2023).

## 7 Future directions and challenges

In addressing the identified limitations and increasing the scientific robustness of research in this domain, several key directions for future studies are proposed. First, large human cohorts encompassing comprehensive data on both the gut microbiome and PTSD, along with an array of potential host factors such as demographic, socioeconomic, and health variables, are recommended. Second, to detect dynamic changes in the microbiota of patients with PTSD, longitudinal assessments rather than relying solely on single stool samples are recommended. Furthermore, initiating these longitudinal studies prior to trauma exposure is crucial for obtaining a comprehensive understanding. For instance, in a war scenario, evaluating soldiers’ predeployment allows monitoring of their microbiota and other biological markers over time, enabling the examination of PTSD development in conjunction with these factors. Similarly, in the general population, studying birth cohorts followed up for many years permits the evaluation of PTSD progression alongside the microbiome and other biological signatures. This approach is essential for disentangling causes and consequences in human studies of PTSD. Third, we recommend the use of whole-metagenome shotgun (WMS) sequencing data to obtain comprehensive taxonomic and functional profiles of the gut microbiome. Metagenomic sequencing offers a more detailed understanding of microbial communities, enabling clinicians to identify specific microbial functions and pathways associated with PTSD. This knowledge could inform targeted interventions aimed at modulating the gut microbiome to improve PTSD outcomes. Fourth, the integration of multiomics data, including metagenomic, metatranscriptomic, and metabolomic data, is proposed to systematically elucidate the mechanisms linking the intestine and PTSD, considering previous findings associating abnormal intestinal conditions with PTSD.

Furthermore, there is an emphasis on enhancing causal inference through novel computational/statistical methods to identify potential PTSD-associated species and pathways. This approach facilitates the rational design of synbiotics (combinations of specific probiotics and prebiotics) for the potential amelioration of PTSD symptoms. Finally, a combination of standardized and varied animal models could be used to explore the relationships between stress-related behavioral changes and the gut microbiome. These proposed directions have three significant implications for future research and practice. First, these findings signify a significant progression by offering insights into whether there are differences in the gut microbiome between individuals with PTSD and those who have experienced trauma alone. This analysis must be conducted with meticulous consideration of various potential host factors. Furthermore, recent findings indicate that PTSD is linked to impaired immune and inflammatory function, various chronic illnesses, and cognitive impairments, all of which have been connected to an altered microbiome. This connection underscores the importance of further research into the role of the gut microbiome in PTSD. Understanding this relationship could lead to new therapeutic strategies, such as microbiome-targeted treatments, to improve immune and inflammatory responses and potentially alleviate some of the chronic conditions and cognitive symptoms associated with PTSD.

## Author contributions

PP: Visualization, Writing – original draft. KD: Writing – review & editing. VO: Writing – review & editing. PB: Writing – review & editing. OK: Supervision, Writing – review & editing.
